# A common neonicotinoid pesticide, thiamethoxam, alters honey bee activity, motor functions, and movement to light

**DOI:** 10.1038/s41598-017-15308-6

**Published:** 2017-11-09

**Authors:** S. Tosi, J. C. Nieh

**Affiliations:** 1 University of California, San Diego, Division of Biological Sciences Section of Ecology, Behavior, and Evolution 9500 Gilman Drive, MC0116, La Jolla, CA 92093-0116 United States; 2 Alma Mater Studiorum, University of Bologna, Department of Agricultural Sciences, Viale Fanin 42, 40127 Bologna, Italy

## Abstract

Honey bees provide key ecosystem services. To pollinate and to sustain the colony, workers must walk, climb, and use phototaxis as they move inside and outside the nest. Phototaxis, orientation to light, is linked to sucrose responsiveness and the transition of work from inside to outside the nest, and is also a key component of division of labour. However, the sublethal effects of pesticides on locomotion and movement to light are relatively poorly understood. Thiamethoxam (TMX) is a common neonicotinoid pesticide that bees can consume in nectar and pollen. We used a vertical arena illuminated from the top to test the effects of acute and chronic sublethal exposures to TMX. Acute consumption (1.34 ng/bee) impaired locomotion, caused hyperactivity (velocity: +109%; time moving: +44%) shortly after exposure (30 min), and impaired motor functions (falls: +83%; time top: −43%; time bottom: +93%; abnormal behaviours: +138%; inability to ascend: +280%) over a longer period (60 min). A 2-day chronic exposure (field-relevant daily intakes of 1.42–3.48 ng/bee/day) impaired bee ability to ascend. TMX increased movement to light after acute and chronic exposure. Thus, TMX could reduce colony health by harming worker locomotion and, potentially, alter division of labour if bees move outside or remain outdoors.

## Introduction

Pollinators provide essential ecosystem services, and the honey bee, *Apis mellifera* L., 1758, is a major global pollinator of crops and native plants^1^. Honey bees are therefore essential for biodiversity conservation, crop production and human welfare^1,2^. However, multiple factors, including diseases, parasites, habitat loss, and pesticides impair honey bee health^3^. As agricultural pollinators, honey bees are routinely exposed to a wide variety of pesticides that reduce colony health^4–6^. Neonicotinoid pesticides are of particular concern because they are neurotoxic insecticides that are used globally on multiple crops visited by honey bees to collect food resources^7–9^.

Neonicotinoids comprise about one third of the insecticides on the global market^9–11^. Although their use has been temporarily restricted in the European Union^12^, they remain commonly used worldwide^13^. Thiamethoxam (TMX) is one of the most widely used neonicotinoids because TMX and its degradation products are highly toxic to insects^9,14^. Since TMX is systemic (spreading to all plant tissues) and environmentally persistent, it is found in multiple resources that bees collect and consume: nectar, pollen, guttation droplets, and water runoff^15,16^. TMX acts by binding with high affinity to insect nicotinic acetylcholine receptors^9^ and thereby elicits a variety of sublethal effects on bees and colonies, even at low doses and concentrations^16^.

We focused on how TMX may impair movement to light and locomotion because of the important role that these behaviours play in colony life and colony division of labour. Young bees are negatively phototactic^17^ and are typically found inside the dark hive, while foragers are positively phototactic^18^ and typically remain either in the proximity of the colony entrance or forage outside the dark hive, even showing a preference for brighter foraging areas^19,20^. Ben-Shahar^21^ showed that a honey bee foraging gene (*amfor*) encodes a cGMP-dependent protein kinase that controls bee positive phototaxis. Upregulation of this gene is associated with the age-related transition from in-hive to outside-hive tasks (i.e. foraging)^21^. Movement to light therefore plays a role in foraging activity and division of labor^22^ because bees transition from tasks inside to outside the hive as they age^20^. The biogenic amines, serotonin^23^, octopamine, and tyramine^24^ also modulate phototaxis. Because these biogenic amines modulate a variety of other behaviours and physiological states^23^, phototaxis can also be an indicator of bee sensitivity to other stimuli, such as nectar sugar concentration. For example, bee responsiveness to sucrose is positively correlated to responsiveness to light^25–27^.

Bees require coordinated walking and climbing as they move inside and outside the nest. Inside the nest, foragers walk and climb on combs and recruit by dancing^28^, which necessitates coordinated locomotion^29^. Outside the nest, they can walk upon inflorescences to obtain nectar and pollen and must also use coordinated leg motions to collect pollen and resin^20^. The effects of pesticides on bee walking have been assessed in different ways (in small petri dishes^30,31^ or in larger, artificially illuminated arenas^32–35^) using different exposure methods (oral^32,33,36^ vs. contact^32–36^ application, acute^32,34–36^ vs. chronic^33^ exposure). However, our understanding of the effects of neonicotinoids such as TMX on bee locomotion and movement to light remain relatively limited.

Different compounds can disrupt honey bee movement to light. Thymol (an essential oil used by beekeepers to control *Varroa*) reduces bee attraction to light (10–4300 ng/bee^37–39^). Pesticides can also impair movement to light and motor control. Teeters *et al*.^30^ measured the distance travelled by walking young workers inside Petri dishes and the amount of time spent in a food zone. They showed that sublethal doses of tau-fluvalinate (a pyrethroid, 0.3–3 µg/bee, contact) and imidacloprid (a neonicotinoid, 50–500 ppb, oral) reduced distance moved and time spent at food source. Medrzycki *et al*.^40^ studied the effects of oral acute exposures to imidacloprid (100–500 ppb) on the time spent walking, stationary or running, and showed that it reduced locomotion. Lambin *et al*.^35^ demonstrated that imidacloprid would impair honey bee motor activity in a similar light-orientation assay (1.25–20 ng/bee, contact). Williamson *et al*.^31^ studied the effects of various neonicotinoids on the walking of bees inside petri dishes and observed that those chronically exposed to imidacloprid, TMX, and clothianidin (10 nM) for 24 h lost postural control, fell over, and were unable to right themselves. Charreton *et al*.^34^ showed that acute contact exposure to TMX (3.8 ng/bee) decreased the time young honey bees spent moving in a vertical arena towards the light. To date, Charreton *et al*.^34^ is the only study that has demonstrated that TMX can impair honey bee movement to light, although at a relatively high dose of 3.8 ng/bee (contact exposure). Hassani *et al*.^32,36^ tested the effect of oral- and contact-administered sublethal doses of fipronil (phenylpyrazole, 0.1–1 ng/bee), acetamiprid and TMX (0.1–1 ng/bee) on the geotaxis and light-orientation of walking workers. They measured total distance walked, duration of immobility, number of ascents and time spent in each of the six levels of the apparatus 60 min after treatment, and showed that acetamiprid (0.1 and 0.5 µg/bee, contact) increased walking. However, they found no effects of TMX and fipronil on walking orientation to light^32,36^. Likewise, Aliouane *et al*.^33^ tested the effects of contact and oral chronic exposure over 11 days to acetamiprid (0.1, 0.5, 1 µg/bee) and TMX (0.1, 0.5, 1 ng/bee) on honey bee walking. They assessed the same parameters as Hassani *et al*.^32,36^, and also observed no significant effect of these neonicotinoids. Thus, the effects of field-realistic exposure to TMX on bee movement to light and locomotion remain unclear. We therefore tested the hypothesis that acute and chronic field-realistic doses of TMX can impair honey bee locomotion and movement to light. In foragers, we examined 11 behavioural parameters related to the locomotion activity, motor functions and movement to light.

## Results

We tested bees in a standard phototaxis arena, a vertical chamber illuminated from above. In our experiments, control bees generally walked directly up the walls and reached the light in 38 ± 5 s, over the course of the 3-min trial. They tended to stay at the top of the arena, directly under the light for 98 ± 7 s. However, depending upon the type of TMX exposure (acute or chronic) and the time after exposure (30 or 60 min after acute exposure), bees exhibited locomotor deficits that included falling and abnormal movements. For simplicity, the detailed statistical results on how TMX affects forager locomotion and movement to light after acute and chronic exposures are shown in Table 1. We provide *p*-values for the results below, with a percentage (+/−) that indicates the direction of each significant effect.

**Table 1:** Summary of the statistical results of the acute and chronic experiments.

Pesticide Exposure	Measure	Colony effect (%)	Factor	DF numerator	DF denominator	L-R *χ* ^2^	*P*-value
Acute	Time_first path_ to reach the light (s)	<1	TMX	1	29	4.90	**0.035**
			Time	1	29	7.98	**0.009**
			TMX × Time	1	29	0.25	0.622
	Distance_first path_ to reach the light (s)	<1	TMX	1	27	0.87	0.359
			Time	1	29	0.00	0.969
			TMX × Time	1	29	0.22	0.645
	Velocity_first path_ to reach the light (squares/s)	<1	TMX	1	29	14.19	**0.001**
			Time	1	29	11.93	**0.002**
			TMX × Time	1	29	1.35	0.255
	Time moving (s)	18	TMX	1	35	5.59	**0.024**
			Time	1	37	0.58	0.450
			TMX × Time	1	37	2.47	0.124
	Velocity (squares/s)	19	TMX	1	35	2.74	0.107
			Time	1	37	0.26	0.613
			TMX × Time	1	37	5.11	**0.030**
	Falls (n)	3	TMX	1	35	6.85	**0.013**
			Time	1	37	1.32	0.258
			TMX × Time	1	37	0.01	0.912
	Time at the top (s)	27	TMX	1	35	7.29	**0.011**
			Time	1	37	5.70	**0.022**
			TMX × Time	1	37	1.19	0.282
	Time at the bottom (s)	30	TMX	1	35	8.06	**0.008**
			Time	1	37	13.00	**0.001**
			TMX × Time	1	37	4.92	**0.033**
	Abnormal behaviours (n)	*	TMX	1	7	4.09	**0.043**
			Time	1	7	4.63	**0.031**
			TMX × Time	1	7	0.23	0.632
	Inability to ascend (n)	*	TMX	1	7	9.17	**0.002**
			Time	1	7	4.63	**0.031**
			TMX × Time	1	7	0.73	0.392
	Inability to reach the light (n)	*	TMX	1	7	3.15	0.076
			Time	1	7	3.43	0.064
			TMX × Time	1	7	1.30	0.255
Chronic	Time_first path_ to reach the light (s)	2	TMX Daily dose	1	27	2.64	0.116
	Distance_first path_ to reach the light (sq.)	<1	TMX Daily dose	1	27	5.10	**0.032**
	Velocity_first path_ to reach the light (sq./s)	4	TMX Daily dose	1	27	0.06	0.814
	Time moving (s)	44	TMX Daily dose	1	31	0.85	0.364
	Velocity (sq./s)	14	TMX Daily dose	1	33	0.06	0.802
	Falls (n)	<1	TMX Daily dose	1	19	1.52	0.232
	Time at the top (s)	29	TMX Daily dose	1	32	0.03	0.867
	Time at the bottom (s)	13	TMX Daily dose	1	33	0.14	0.709
	Abnormal behaviours (n)	*	TMX Daily dose	1	4	2.00	0.158
	Inability to ascend (n)	*	TMX Daily dose	1	4	4.38	**0.036**
	Inability to reach the light (n)	*	TMX Daily dose	1	4	0.08	0.784

Foragers were tested twice after acute exposure (30 and 60 min), and once after chronic exposure (60 min). Variance component estimates (REML algorithm) of colony effect could not be assessed (*) for the nominal parameters. Acute exposure: *N*_control_ = 19, *N*_TMX_ = 20 (continuous measures, Repeated-Measures ANOVA_REML_ Repeated Measures); *N*_control_ = 19, *N*_TMX_ = 20 (nominal measures, Multiway Frequency); *N*_control_ = 18, *N*_TMX_ = 13 (first path measures, Repeated-Measures ANOVA_REML_ Repeated Measures). Chronic exposure: *N*_control_ = 21, *N*_TMX_ = 15 (continuous measures: ANCOVA_REML_; nominal measures: Nominal Logistic regression); *N*_control_ = 18, *N*_TMX_ = 12 (first path measures, ANCOVA_REML_). Significant effects (*p* < 0.05) are shown in bold.

### Acute exposure

#### TMX increased velocity of the first path towards the light

**Figure 1:**
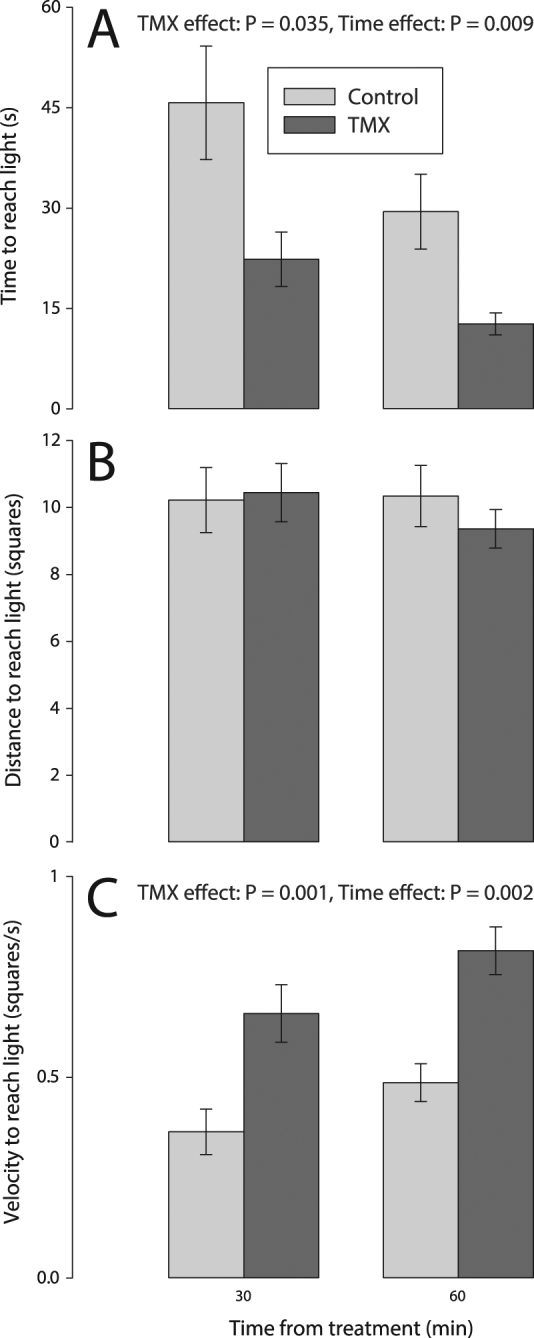
Sublethal effects of TMX on movement to light measured 30 min and 60 min after acute exposure. We show the (**A**) time_first path_, (**B**) distance_first path_ and (**C**) velocity_first path_ to reach the light. Main effects of TMX, time and TMX× time interaction are reported only if significant (see Table 1 for details, ANOVA_REML_ Repeated Measures, *N*_control_ = 18, *N*_TMX_ = 13). The analysis of the parameters related to the first path towards the light included only bees that managed to reach the light. The *p*-values of significant main effects are shown within each plot. Error bars show standard errors.

Acute exposure to TMX caused bees to move more rapidly, as compared to control bees, during their first path to the light. TMX increased bee velocity_first path_ (+61%, *p* = 0.001) and reduced the time_first path_ (−52%, *p* = 0.035) that bees took to reach the light during their first path to the light (Fig. 1, Table 1). There was no effect of TMX on distance_first path_ to reach the light (*p* = 0.36).

There was an effect of time (30 vs. 60 min after treatment) on the velocity_first path_ towards the light (+36% higher velocity at 60 min, *p* = 0.002) and time_first path_ to reach the light (−35% less time at 60 min, *p* = 0.009) (Fig. 1). There was no significant effect of time on distance_first path_ to reach the light (*p* = 0.97) or the interaction TMX× time on distance_first path_, time_first path_ or velocity_first path_ to reach the light (*p* > 0.26, Fig. 1).

#### TMX increased shorter-term hyperactivity

**Figure 2:**
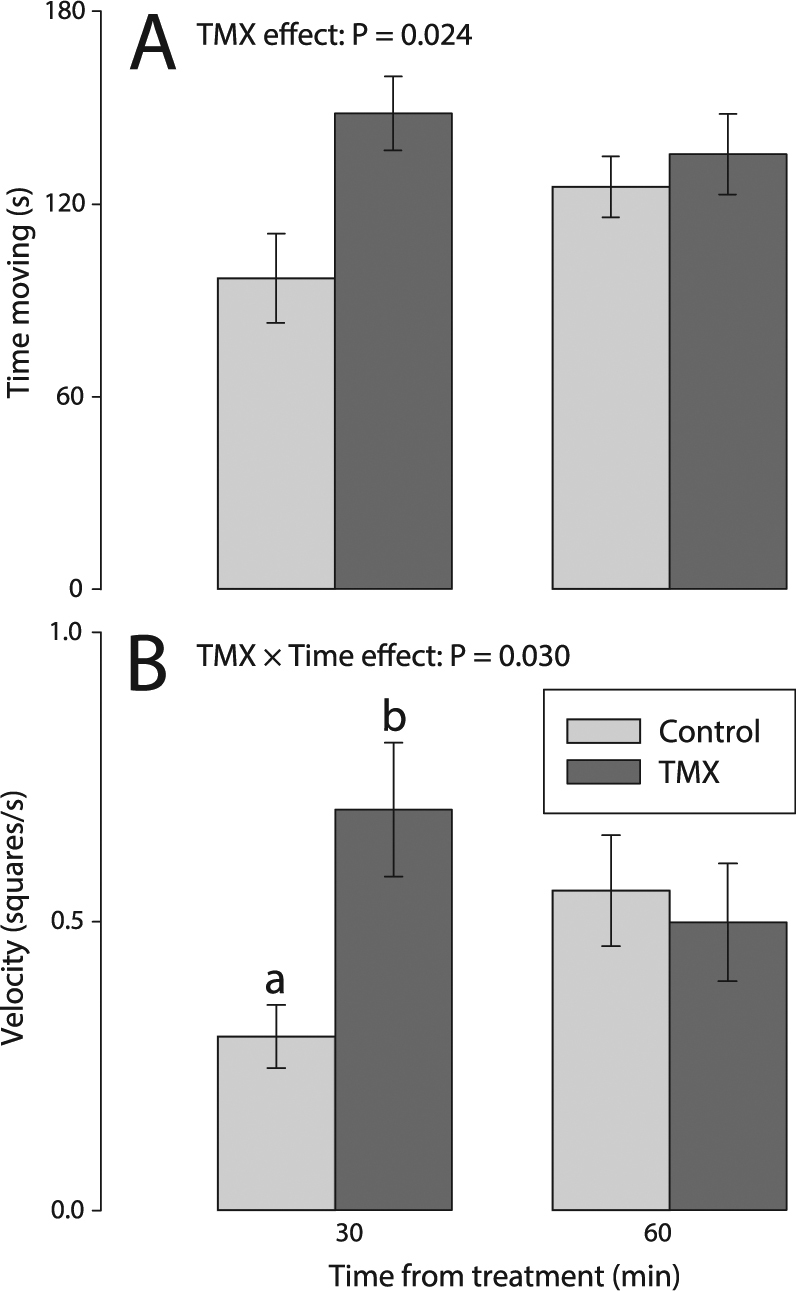
Sublethal effects of TMX on forager activity behaviours measured 30 min and 60 min after acute exposure: (**A**) time spent moving and (**B**) velocity. Main effects of TMX, time and TMX × time interaction are reported only if significant (see Table 1 for details, ANOVA_REML_ Repeated Measures, *N*_control_ = 19, *N*_TMX_ = 20). The *p*-values of significant main effects are shown within each plot. Different letters indicate statistically significant differences after post-hoc LS Means contrast tests. Error bars show standard errors.

Acute exposure to TMX increased bee activity over the 3-min trial, primarily 30 min after treatment. There were no effects of TMX or time (30 vs. 60 min) on the velocity of the bees (*p* > 0.11, Fig. 2, Table 1). However, there was an effect of the interaction TMX× time on bee velocity (*p* = 0.030). TMX increased bee velocity 30 min after treatment (+109%, LS Means contrast test, *F*_1,66_ = 7.22, *p* = 0.0091), but there was no effect of TMX at 60 min (contrast test, *F*_1,66_ = 0.004, *p* = 0.9531, Dunn-Sidak correction: *k* = 2, *α* = 0.0253), as compared with control bees (Fig. 2).

TMX increased the time spent moving (+28%, *p* = 0.024, Fig. 2). There were no effects of time and the TMX× time interaction on time spent moving (*p* > 0.12). Although there was no overall interaction of TMX× time, TMX increased the amount of time that bees spent in motion, as compared to controls, 30 min after exposure (contrast test, *F*_1,62_ = 8.06, *p* = 0.006).

#### TMX reduced longer-term motor functions

**Figure 3:**
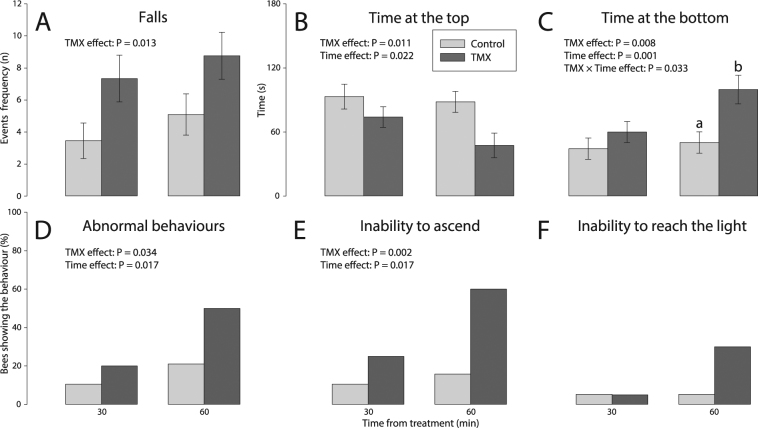
Sublethal effects of TMX on forager motor functions measured 30 min and 60 min after acute exposure. We show the (**A**) number of falls per bee, (**B**) time spent at the arena top, and (**C**) time spent at the arena bottom (ANOVA_REML_ Repeated Measures, N_control_ = 19, N_TMX_ = 20). We also plot the frequencies of bees showing (**D**) abnormal behaviours (see Methods for definitions), (**E**) inability to ascend the arena, and (**F**) inability to reach the light (Multiway Frequency, *N*_control_ = 19, *N*_TMX_ = 20). The *p*-values of significant main effects are shown within each plot. Main effects of TMX, time and TMX× time interaction are reported only if significant (see Table 1 for details). Different letters indicate statistically significant differences after post-hoc LS Means contrast tests. Error bars show standard errors.

Over the 3-min trial, acute exposure to TMX impaired various bee motor functions, primarily 60 min after treatment. Overall, TMX increased the number of falls (+92%, *p* = 0.013) and the time at the bottom of the arena (+67%, *p* = 0.008). TMX also reduced the time spent at the top of the arena (−32%, *p* = 0.011) (Fig. 3, Table 1). TMX increased the number of bees that showed abnormal behaviours (+90%, *p* = 0.034) or exhibited inability to ascend (+209%, *p* = 0.002), as compared to control bees (Fig. 3). There was no effect of TMX on bee ability to reach the light (*p* = 0.08).

There was an effect of time on time at the top (*p* = 0.022) and at the bottom (*p* = 0.001) of the arena, frequency of abnormal behaviours (*p* = 0.031) and inability to ascend (*p* = 0.031) (Fig. 3). There was no effect of TMX on bee ability to reach the light (*p* = 0.06). The times spent at the top and at the bottom were more strongly altered at 60 min (−43%, +93%, respectively) as compared to 30 min (−21%, +36%, respectively) for TMX treated bees (Fig. 3). Similarly, more TMX-treated bees showed abnormal behaviours and inability to ascend at 60 min (+138% and +280%, respectively) as compared to 30 min (+90% and +138%, respectively) (Fig. 3).

There was an effect of the interaction TMX× time on time spent at the bottom of the arena (*p* = 0.033). TMX increased the time spent at the bottom at 60 min after treatment (+93%, contrast test, *F*_1,61_ = 12.89, *p* = 0.0007), but there was no effect at 30 min (contrast test, *F*_1,62_ = 1.31, *p* = 0.26, Fig. 3).

### Chronic exposure

#### TMX decreased distance covered to reach the light for the first time

**Figure 4:**
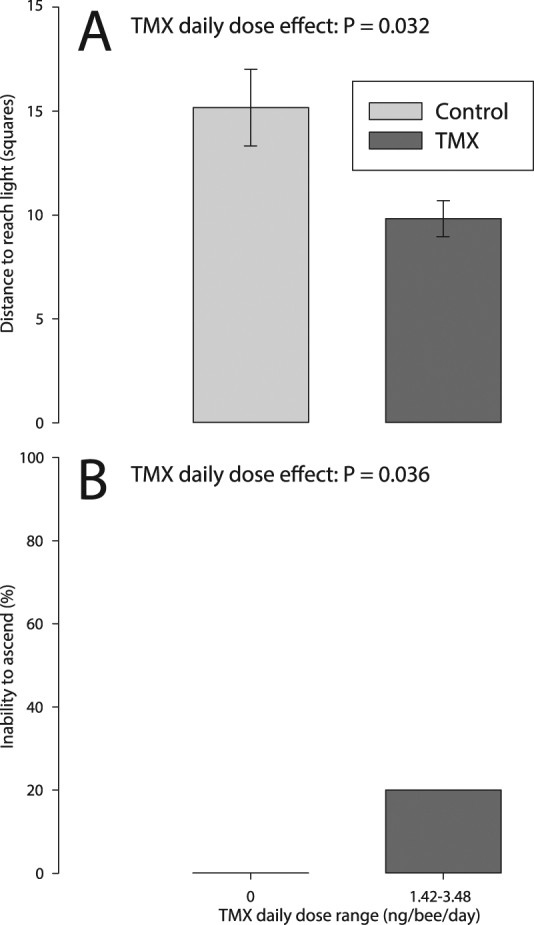
Sublethal effect of chronic exposure to TMX (Range_TMX daily doses_ = 1.42–3.48 ng/bee/day) on (**A**) distance travelled to reach the light and (**B**) inability to ascend. The *p*-values of significant main effects are shown within each plot (see Table 1 for details, **A**: ANCOVA_REML_, *N*_control_ = 18, *N*_pesticide_ = 12; **B**: Nominal Logistic, *N*_control_ = 21, *N*_pesticide_ = 15).

During the first path towards the light, chronic exposure to TMX resulted in bees taking a shorter path to reach the light (distance_first path_ to reach the light: −35%, *p* = 0.016, Fig. 4, Table 1). There was no effect of TMX on time_first path_ (*p* = 0.13) and velocity_first path_ (*p* = 0.77) to reach the light.

#### TMX reduced the ability of the bees to climb

Over a 3-min trial, chronic exposure to TMX increased the proportion of bees unable to ascend the arena (20% vs 0%, *p* = 0.021, Fig. 4, Table 1). There was no significant effect of TMX on the number of falls (*p* = 0.15), time at the bottom (*p* = 0.46), time at the top (*p* = 0.46), proportion of bees showing abnormal behaviours (*p* = 0.32), or inability to reach the light (*p* = 0.62) (Fig. 4).

#### TMX reduced sucrose consumption the first day of incubation

During the 2-day incubation period, chronic exposure to TMX reduced sucrose consumption on the first day (−19%, Kruskal-Wallis Rank Sums, *χ* ^2^ = 3.96, *p* = 0.0466). There was no effect of TMX on 2-day cumulative sucrose consumption (*χ* ^2^ = 2.01, *p* = 0.16) or average daily sucrose consumption (*χ* ^2^ = 1.32, *p* = 0.25, Kruskal-Wallis Rank Sums).

#### No significant effect of TMX on activity

Over the 3-min trial, there was no effect of chronic TMX exposure on time spent moving (*p* = 0.23), distance covered (*p* = 0.94), or overall velocity (*p* = 0.94) of bees (Fig. 4, Table 1).

## Discussion

We present the first evidence that acute and chronic oral TMX exposure at field-realistic, sublethal levels can significantly alter forager movement to light. Locomotion is essential for flight and foraging ability, and its impairment could therefore have an impact on the quality of pollination services provided by the bees. Henry *et al*.^41^ used the same single oral dose of TMX that we used and showed that this sublethal intoxication significantly reduced foragers homing success. Their results were further confirmed by subsequent studies showing that similar TMX field-realistic doses altered homing success and colony health^42^ and the physical ability of foragers to fly^43^.

We show that the effects of TMX on motor function and activity varied over time, but the effects on immediate attraction to light were consistent over time and exposure mode. Essentially, treated bees became hyperactive (abnormally active) 30 min after acute exposure, but this effect disappeared in the longer term, either 60 min after acute exposure or following chronic exposure. Shortly after acute exposure (30 min), neonicotinoid-treated bees moved significantly faster (+109%) and spent significantly more time moving (+44%) than control bees (Table 1, Fig. 2). However, after 60 min, TMX impaired motor functions, reducing the ability of foragers to walk and to climb (Fig. 3). TMX-treated foragers fell more often (+83%), exhibited more abnormal behaviours (+138%), and were more frequently unable to ascend the arena (+280%). These bees also spent more time at the bottom (+93%) and less at the top (−43%) of the arena, as compared to control bees. Immediate attraction to light increased with time after acute exposure (30 vs. 60 min), possibly because foragers were more familiar with the arena during their second exposure to it.

Our TMX chronic exposure led to field realistic daily doses (Range_TMX daily doses_ = 1.42–3.48 ng/bee/day), which reduced the ability of foragers to ascend the arena walls, but had no significant effect on the other locomotion measures. Williamson *et al*.^31^ observed that 1 day of TMX exposure (10 nM) reduced postural control in petri dishes (increased the time that bees were flipped upside down), while Aliouane *et al*.^33^ found no significant effects on locomotion of bees observed in arena after 11-days of multiple daily exposure to TMX doses (0.1 and 1 ng/bee, oral and contact). Similarly, 1 or 2 days of exposure to TMX (Range_TMX daily doses_ 1.26–4.53 ng/bee/day) impaired forager motor functioning by reducing flight duration, distance, mean velocity, and maximum velocity^43^.

Phototaxis is defined as movement to light, but it is possible that bees would also move in our apparatus in the absence of light. Thus, we cannot disentangle the effects of phototaxis from locomotion alone. Future studies that compare locomotion in the presence and absence of light^44^ would enable researchers to decouple the effects of neonicotinoids on phototaxis and locomotion. However, our data show that TMX can differentially affect attraction to light and locomotion. For example, 60 minutes after acute exposure, TMX-treated bees showed a decreased ability to walk and climb (e.g. increased frequency of abnormal behaviours and falls, increased time spent at the bottom of the arena, Fig. 3), but they still moved towards the light and reached it faster than control bees (Fig. 1). Similarly, after chronic exposure, TMX-treated bees showed a reduced motor ability (perhaps because of neuro-motor impairments, lack of energy, or both), but were still more attracted to light (Fig. 4). Thus, our results show that chronic and acute (60 min after treatment) exposure to TMX increased bee movement to light even when their locomotion ability was reduced. TMX-treated bees were more active and reached the light faster when tested 30 min after acute exposure.

Our results support those of prior studies. Activity parameters, such as time moving and velocity, were not significantly altered 60 min after an acute TMX treatment, matching the results from Hassani *et al*.^36^. They also found no significant effect of TMX (oral and contact) on the total distance walked by worker bees, duration of immobility, and number of ascents (from one level of the arena to another) 60 min after treatment. Our results showed that TMX impaired motor functions in the longer term, confirming the results of Charreton *et al*.^34^. Charreton *et al*.^34^ showed that TMX (3.8 ng/bee, acute contact) reduced the distance walked in an arena by newly emerged bees, 4–8 hrs after treatment. Likewise, we recently demonstrated that TMX exposure to the same acute dose (1.34 ng/bee) and to similar chronic doses (Range_TMX daily doses_ 1.26–4.53 ng/bee/day) elicited opposite effects on forager flight abilities: bees were excited and flew longer time and distance shortly after acute exposure (40 min), but flew more slowly over shorter distances after longer exposures (1 and 2 days)^43^.

Our TMX results are also consistent with the demonstrated effects of other neonicotinoids. Imidacloprid (0.1–1000 ng/bee, acute exposure) elicited opposite effects on walking ability depending upon time from exposure. Specifically, imidacloprid rapidly led to hyperactivity that later disappeared^40,45–47^. Bees fed imidacloprid (2.5 ng/bee, acute oral) spent less time stationary in the shorter term (15 min) and were more stationary in the longer term (60 min^35^). Similar effects occurred with German cockroaches (*Blattella germanica* L., 1767), which showed hyperactivity rapidly after an imidacloprid treatment, and slower behaviours, hypoactivity, later^48^. The neonicotinoid acetamiprid (100 and 500 ng/bee) elicited hyperactivity on worker bees after acute contact application, increasing the distance covered and decreasing immobile time, though these results were not confirmed after oral applications^36^.

Increased movement to light caused by TMX should lead bees outside of the dark nest. Rueppel *et al*.^49^ showed that bees poisoned with hydroxyurea (a cytostatic drug used for human chemotherapy) tended to leave the nest to die, a phenomenon that is also observed in *Varroa*-infested bees, and is considered a form of social immunity since it reduces nestmate exposure to harmful compounds or parasites^50,51^. Likewise, increased movement to light resulting from TMX could increase the propensity of foragers to remain outside the nest and thereby reduce nestmate and colony contamination, though this prediction remains to be tested. The neurons and biogenic amines that modulate movement to light are involved in a variety of other behaviours and physiological states^23,24^. In fact, phototaxis is positively correlated with bee sensitivity to other stimuli, such as nectar sweetness (gustatory responsiveness)^25,26^. Movement to light is also related to the transition from in-hive to outside-hive tasks and is involved in other complex social behaviours^21,27,52–56^. Thus, alterations of bee movement to light could have broad colony fitness effects, changing foraging activity (performing recruitment dances, walking and climbing on the combs) and division of labour.

## Methods

This study was conducted at University of California San Diego (UCSD), Division of Biological Sciences (La Jolla, CA, USA). We used six healthy honey bee colonies (*A. mellifera ligustica* Spinola, 1806, 10 frames per colony) housed at the UCSD Biology Field Station apiary. We tested a total of 78 bees.

### The arena

**Figure 5:**
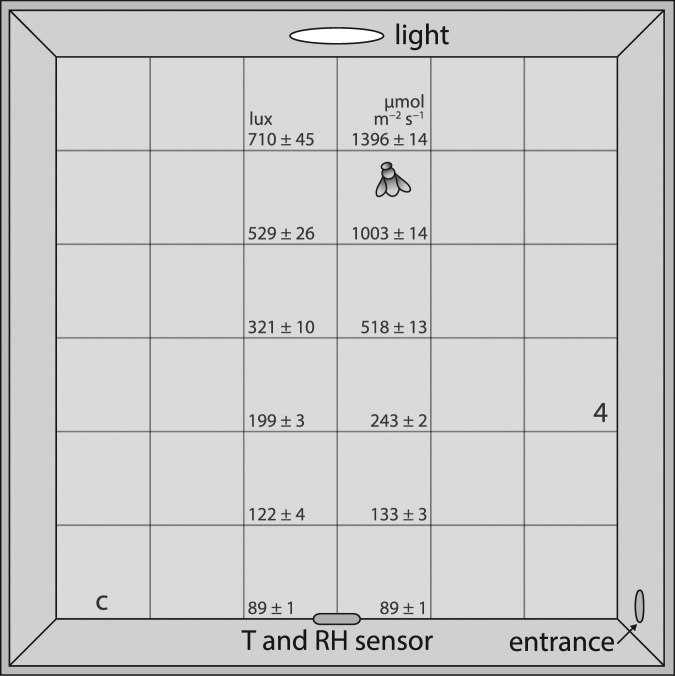
The arena used to test activity, motor functions, and movement to light of foragers. A bee is drawn approaching the light near the top of the arena. The amount of light (left: luminous flux (lux); right: Photosynthetic Photon Flux Density (µmol m^−2^ s^−1^); mean ± standard error) at the centre of the arena, directly below the light, is shown for each level. The oval at the base of the arena shows the position of the temperature (T) and relative humidity (RH) sensor.

We used a vertical arena illuminated from the top (Fig. 5, 30 × 30 × 5 cm) to assess forager locomotion and movement to light (Fig. 5). The arena was designed so that it would match a normal vertical comb environment. Previous studies tested bee walking through smaller spaces (petri dishes: 9 cm^30^; 15 × 1.5 cm^31^) or a similar apparatus (also vertical and artificially illuminated from above, 30 × 30 × 4 cm^32–35^). Our arena had white acrylic side walls and transparent acrylic back, front and top. The front could slide open to allow bee removal and cleaning after each test; a grid of white paper (36 squares, 5 cm in width) was placed on the outside of the back wall for measurements. The interior was completely composed of plastic to facilitate cleaning. Bees walked through a 1 cm diameter tubular entrance at the bottom right of the arena. We divided the arena in 36 cubes (Fig. 5, 5 × 5 × 5 cm) that we used to measure the location of the bee during the test. A LED light (luminous flux: 280 lm, 120° illumination angle, 6000 K color temperature) was centred at the top and pointed to the bottom (Fig. 5). During trials, no other lights were used. Light intensity was maintained at a constant 280 lm, because light intensity influences the phototactic behaviour of the bees^25,26^. We used a light intensity of 280 lm because this level of brightness provided a sufficient phototactic effect, attracting bees who travelled from the bottom towards the light within the trial duration of 3 min.

Because the light levels inside the arena varied depending on the distance from the light source, we quantified the light levels (level 1–6, 10 replicates per each level) using a digital Lux meter (Dr. Meter, model LX1330B, measuring range of 0.1~200,000 Lux, resolution of 0.1 Lux, Fig. 5). To measure the Photosynthetic Photon Flux Density (PPFD, defined as the number of photons in the photosynthetic range received by a 1 m^2^ surface per s) we used a Photosynthetically Active Radiation (PAR) sensor (Vernier Software & Technology, PAR range 0–2000 µmol m^−2^ s^−1^, resolution 1 µmol m^−2^ s^−1^, spectral range 410–655 nm, Fig. 5). The wavelength range of this standard sensor is somewhat similar to the spectral range of honey bee vision (300–650 nm)^57,58^. In our apparatus, 280 lm corresponded to an illuminance range_bottom-top_ of 89–710 lux and a PPFD range_bottom-top_ of 89–1396 µmol m^−2^ s^−1^ (Fig. 5). These light levels span the intensities (14–560 lux^25–27,34^) used in previous phototactic experiments, and the range of light at our apiary (in shade (N = 10): 97 ± 1 µmol m^−2^ s^−1^; in full sun (N = 10): 1646 ± 26 µmol m^−2^ s^−1^).

The arena included a temperature and relatively humidity (RH) sensor (Fig. 5). Temperature and RH were at 25 ± 1 °C and between 50–80% RH during the experiments. We placed the video camera (Sony, model NXCAM Exmor R) directly in front of and perpendicular to the plane of the arena.

### Honey bee preparation

We wished to study foragers, the bees most likely exposed to TMX, and therefore focused on bees returning to the nest with pollen in their corbiculae since these bees are, by definition, foragers. Returning pollen foragers were individually captured in vials at hive entrances. After collection, foragers were placed into plastic cages (11 × 11 × 9 cm, 10 bees/cage) and maintained in an incubator at 30 ± 1 °C and 50–80% RH with sucrose solution *ad libitum* for 1 h for the acute experiment or 48 h for the chronic experiment.

### Forager behaviours in the Arena

To test the effect of TMX over time, each forager was tested inside the arena twice (30 min and 60 min after treatment). During each test, bee behaviours were recorded for 3 min, as in similar studies^32–34^. We chose this time interval because 3 min was more than sufficient for control bees to reach the light. To begin each trial, a bee was carefully captured from a plastic cage into a plastic vial that was then gently placed over a 1 cm long tube at the bottom, right side of the arena (Fig. 5). The room was completely dark, with the only light coming from the LED bulb at the top of the arena (Fig. 5). Bees then instinctively moved towards the light by entering the arena and then climbing up^33^. After reaching the light, bees (particularly those treated with TMX) would sometimes fall and could then climb again towards the light. Over the 3-min observation period, we measured 11 behavioural parameters (definitions in Table 2) related to bee activity, motor function and movement to light. Three parameters were related to the first path towards the light, which starts from the moment the bee enters the arena until the moment the bee reaches the light (time_first path_ spent to reach the light for the first time, distance_first path_ of the first path towards the light, and velocity_first path_ during the first path towards the light). The other eight parameters referred to the whole 3-min period (overall velocity, time spent moving, time spent at the top, time spent at the bottom, overall number of falls, inability to reach the light, inability to ascend the arena walls, and exhibiting abnormal behaviours, Table 2). We calculated bee velocity by dividing the distance walked (number of 5 × 5 cm squares crossed) by time.

**Table 2:** List and definitions of parameters assessed during the phototaxis arena tests.

Measure	Definition
Time_first path_ to reach the light (s)	Time spent to reach the light for the first time
Distance_first path_ to reach the light (squares)	Number of squares crossed to reach the light for the first time
Velocity_first path_ to reach the light (squares/s)	Mean velocity during the first path to reach the light
Velocity (squares/s)	Mean overall velocity
Time moving (s)	Time spent moving
Falls (n)	Frequency of falls
Time at the top (s)	Time spent within 5 cm of the top
Time at the bottom (s)	Time spent within 5 cm of the bottom
Inability to reach light (T/F)	The bee can climb partially to reach the light but does not fully succeed even once
Inability to ascend (T/F)	The bee is unable to climb the arena walls for at least 10 seconds
Abnormal behaviours (T/F)	The bee exhibits an abnormal behaviour such as trembling (shaking/shivering of body, legs or antennae of a bee that is typically twitching or unable to get up), loss of coordination (falls while walking or stumbles), erratic movements (walks in circles and/or in atypical patterns) for at least 10 seconds

A preliminary analysis of a randomly selected sample of our data using video tracking (Tracker v4.96) yielded similar results (time to light: *F*_1,15_ = 6.36, *p* = 0.024; distance to light: *F*_1,14_ = 0.45, *p* = 0.515) as our simplified analysis of movements over squares. Measuring movement over squares facilitated the rapid analysis of the movements and the abnormal behaviours of more bees. It also created a simpler assay with greater potential for widespread use. We therefore use the analysis of movements over squares throughout this paper.

The inability to ascend the arena walls and the presence of general abnormal behaviours (trembling, loss of coordination, erratic movements) were scored if bees exhibited such behaviours for longer than 10 seconds throughout the 3-min test. We define trembling as the shaking or shivering of body, legs or antennae. A bee shows loss of coordination when she falls while walking or stumbles. We defined erratic movements as a bee walking in small circles or in other atypical patterns.

At the end of the trial, we opened the arena, carefully caught the bee in a vial and then thoroughly cleaned the arena with 100% ethanol to remove potential bee odours. We then allowed the arena to fully dry before reusing it.

### Pesticide concentrations and doses

There is a wide range of field-relevant pesticide doses and concentrations, with variations across time and space^59^. Because we exposed foragers by feeding them TMX in sucrose solution, TMX levels in nectar provide the most realistic residue levels. However, we note that in relatively rare cases, foragers can contain higher TMX residues in their tissue (310 ppb^60^) and can consume higher concentrations of TMX (100 ppm^61^) by ingesting guttation droplets produced by TMX seed-treated plants such as corn and oilseed rape^62^.

The acute and chronic experiments and their respective analysis were based on the actual dose of TMX consumed by each bee (average per bee per cage). All TMX doses that we tested were lower than the worst case scenario thresholds, defined using calculations from the European Food Safety Authority (EFSA)^63^. The worst-case scenario calculations considered the field-relevant amount of nectar consumed by foragers, based upon the sucrose content of nectar and their energy requirements during foraging activity, and the highest TMX concentration found in nectar to which bees could be exposed. Because the worst-case scenarios were estimated depending on time of exposure, we compared our acute and chronic doses to calculated acute and chronic scenarios^63^.

In the *acute exposure* experiment, bees were fed a single dose of TMX (1.34 ng, 4.6 pmol). This same dose was used by prior studies that demonstrated an impact of TMX on forager homing^41^ and flight ability^43^. This dose is 3.7 times lower than the LD_50_ of TMX^64^ and does not significantly increase mortality as compared to controls^41^. Although some authors consider 1.34 ng to not be field-relevant^65^, we calculated (based upon EFSA^63^) that foragers can acutely consume up to 1.80 ng TMX/bee in 1 h of foraging for nectar (10% sugar w/w, oilseed rape^66,67^ contaminated with 15 ppb of TMX (transplant-drip application^68^). Thus, 15 ppb^68^ is a fairly high TMX concentration, though even higher concentrations of TMX have been found in nectar by Sanchez-Bayo and Goka (17 ppb^69^), Dively and Kamel (19 ppb, including TMX metabolites^68^), and Stoner and Eitzer (20 ppb^70^). Reviews by Bonmatin *et al*.^15^ and Godfray *et al*.^16^ are informative. Because transplant-drip applications are typically a short-term contamination route for bees, we used this 15 ppb level to calculate the worst case acute exposure scenario (1 h short-term exposure^63^) only. Therefore, we tested an acute sublethal dose that was lower than the worst-case scenario (<1.8 ng/bee/1 h) in which bees foraged for 1 h on nectar that was contaminated by TMX after a transplant-drip application.

In the *chronic exposure* experiment, we exposed bees to a range of TMX daily doses (Range_TMX daily doses_ = 1.42–3.48 ng/bee/day = 4.9–11.9 pmol/bee/day, Mean_TMX daily doses_ = 2.56 ± 0.12 ng/bee/day, *N*_TMX daily doses_ = 4). These daily doses reflected the actual TMX consumption per bee cage, and resulted from feeding bees a sucrose solution containing 45 ppb of TMX. EFSA estimated that foragers could consume up to 6.66 ng TMX/bee/day when foraging nectar from TMX seed-treated plants (i.e. oilseed rape) containing 5 ppb of TMX^63^. In our experiments, foragers consumed TMX daily doses that were always lower than 6.66 ng of TMX/bee/day.

Foragers consume less sucrose per day when maintained in cages as compared to colonies, perhaps because they have reduced activity in small cages. The foragers we reared in the lab consumed 61 ± 10 (control) and 57 ± 15 (TMX) mg/bee/day of sugar, while it’s been estimated that foragers could consume up to 128 mg/bee/day of sugar while foraging in the field^63^. To achieve field-relevant TMX daily doses approaching a realistic worst-case scenario, we provided foragers with a TMX solution that was more concentrated (45 ppb) than what is typically found in nectar after seed treatments. Therefore, we focused our analyses on the field-relevant TMX daily doses consumed by our bees.

We used analytical grade TMX (CAS#153719-23-43, Sigma Aldrich 37924-100MG-R). We prepared the stock solution with double-distilled H_2_0, and we maintained it at 4 °C inside a bottle completely wrapped in aluminium foil to avoid light degradation^15^. We prepared the solutions used to feed the bees daily, by diluting the stock solution with 1.8 M sucrose solution.

### Acute exposure

After collection, foragers were incubated for 1 h with 0.5 M sucrose solution (pesticide-free, prepared with analytical grade sucrose and double-distilled water) *ad libitum*, to allow them to adjust to their new setting and to help equalize their hunger levels. After the 1 h incubation, bees were starved for 30 min to allow them to subsequently consume 10 µl of 1.8 M sucrose test solution. For feeding, each bee was individually inserted in a modified syringe (2.5 mL). During feeding, the plunger was gently pushed to coax the bee to the end of the syringe, in which a 2-mm diameter hole was cut and through which the bee was fed the test solution. The test solution was either pure sucrose or contained the same TMX dose used by Henry *et al*.^41^ and Tosi *et al*.^43^: 1.34 ng (corresponding to 118 ppb, 134 µg/L and 460 nmol/L). For these calculations, we took into account the density of 1.8 M sucrose solution at 20 °C and 1 ATM (1.230 kg/L^71^). After the individual feeding, we placed each bee into a separate cage to prevent food exchange with other bees. We maintained these cages in an incubator at 30 ± 1 °C, 50–80% RH, with no food, for 60 minutes after exposure, excluding the 3-min trial occurring in the arena 30 min after exposure. We tested 42 bees from three colonies.

### Chronic exposure

Bees can be chronically exposed to neonicotinoids if they continue to forage over multiple days at a food source contaminated with the pesticide. We therefore tested the chronic effects of TMX. After collection, foragers were incubated for 2 days with 1.8 M sucrose solution *ad libitum*. The solutions were either pure sucrose or contained 45 ppb of TMX (corresponding to 55 µg/L and 189 nmol/L). The consumption of sucrose solution and TMX per bee was measured daily by weighing syringes. To measure potential evaporative loss, we separately used 10 cages maintained in identical conditions but without bees. We accounted for this evaporative loss (<1%) in our calculations. We tested 36 bees from four colonies.

### Statistical analysis

In the *acute exposure* experiment, we tested bees at 30 and 60 minutes after exposure. We used Repeated-Measures Analysis of Variance (ANOVA) with a REML algorithm to test the effects of pesticide treatment (control vs. TMX) at 30 and 60 min after exposure upon the following continuous measures: number of falls (counts), velocity (squares/s), time at the top (s), time at the bottom (s), time moving (s), distance_first path_ to reach the light (number of squares), time_first path_ to reach the light (s), and mean velocity_first path_ to reach the light (squares/s). Colony was included as a random factor. Significant effects were further analysed with post-hoc Least-Square Means contrast tests. We applied the Dunn-Sidak method^72^ to correct for multiple comparisons. We used a Repeated-Measures Multiway Frequency analysis^73^ to test the effect of pesticide treatment (control vs. TMX), time (30 vs. 60 min post-treatment) and their interaction on the following nominal measures: abnormal behaviours (Y/N), inability to ascend the arena (Y/N), and inability to reach the light (Y/N). All tested bees participated to both arena tests (30 and 60 min after treatment).

In the *chronic exposure* experiment, we tested bees at 60 min after exposure. We used Mixed-Model ANCOVA and tested TMX daily doses as a continuous effect (*N*_*TMX daily doses*_ = 4) and colony as a nominal effect (random grouping variable, REML algorithm) on the same continuous parameters examined in the acute exposure experiment (see above). We used Nominal Logistic regression to test the effect of TMX daily doses (*N*_*TMX daily doses*_ = 4) upon these nominal parameters: abnormal behaviours, inability to ascend the arena, and inability to reach the light. In these Nominal Logistic models, we included colony as a fixed effect. The sucrose consumption data were not normally distributed, and we therefore used the Kruskal-Wallis Rank Sum test to assess the effect of treatment on sucrose consumption.

We used R v3.3.2^74^ and JMP v10.0 statistical software. We also used residuals analyses to confirm the appropriateness of our models. We report mean ± 1 standard error (s.e.m.). We used an alpha value of 0.05, corrected, as necessary, using the Dunn-Sidak method (see above). We only analysed bees that remained alive for the entire arena trial (180 seconds, 99% of bees). The analysis of the parameters related to the first path towards the light included only bees that managed to reach the light. Inability to reach the light was captured by the analysis of the *inability to reach light* variable.

## Electronic supplementary material

Supplementary Dataset S1.

Supplementary Dataset S2.
